# The phylogenomic position of the sabre squirrelfish *Sargocentron spiniferum* (beryciformes: holocentridae) inferred from the mitochondrial genome

**DOI:** 10.1080/23802359.2016.1209099

**Published:** 2016-09-05

**Authors:** Hao Chen, Haolang Zhou, Xin Wang, Xiao Chen

**Affiliations:** a Department of Marine Biotechnology, School of Life Science, Wenzhou Medical University, Wenzhou, Zhejiang, P.R. China;; b Guangxi Key Lab for Mangrove Conservation and Utilization, Guangxi Mangrove Research Center, Beihai, Guangxi, P.R. China

**Keywords:** *Sargocentron spiniferum*, Holocentridae, mitochondrial genome

## Abstract

In this study, the complete mitochondrial genome of the sabre squirrelfish *Sargocentron spiniferum*, one member of family Holocentridae, is determined. It is a circular molecule whose length reaches 16,548 bp, consisting of 37 genes with typical order to most Chinese vertebrates. The nucleotide composition is: 30.3% A, 29.0% C, 15.3% G and 25.5% T. Located in gene junctions, there are total 39 bp short intergenic spaces and 30 bp overlaps. Two initial codons (GTG and ATG) and three terminal codons (TAG, AGG and TAA/T) are used in the protein-coding genes. Twenty-two tRNAs range from 67 to 74 bp. The control region (865 bp) is located between the tRNA-*Pro* and tRNA-*Phe* genes. The phylogenetic results show that *S. spiniferum* is sister to *S. rubrum*.

The sabre squirrelfish *Sargocentron spiniferum*, one member of family Holocentridae, is mainly distributed in Indo-Pacific (Randall & Heemstra 1986) where it inhabits a variety of reef zones from reef flats to lagoon and seaward reefs to a depth of at least 122 m (Lieske & Myers 1994). This nocturnal and solitary species is the largest squirrelfish which feeds on crabs, shrimps and small fishes (Kuiter & Tonozuka 2001). Here, we determine and provide the whole mitochondrial genome of *S. spiniferum*, then construct the phylogenetic tree of Beryciformes.

One specimen of *S. spiniferum* was captured from the South China Sea, China. It was preserved in Guangxi Mangrove Research Center with voucher LY2015040507. The experimental protocol, data analysis followed the methods of Chen et al. (2016). Including *S. spiniferum*, 13 species of Beryciformes with the complete mitogenomes available in the GenBank were selected in Bayesian phylogenetic reconstruction (three partitions: two rRNA genes, the first and second codons of the 12 protein-coding genes (except *ND6* gene)).

Whole mitochondrial genome of *S. spiniferum* is a circular molecule whose length reaches 16,548 bp (Genbank Accession Number: KX254549). It has the same gene composition, arrangement and transcriptional order as the mitogenome of most vertebrates, which means it also contains 13 protein-coding genes, 2 rRNA genes, 22 tRNA genes and a control region. Located in junctions among those genes, there are total 39 bp short intergenic spaces and 30 bp overlaps. The nucleotide base composition of this genome is 30.3% A, 29.0% C, 15.3% G and 25.5% T. Except for *CO1* gene that uses GTG as the initial codon, the remaining 12 protein-coding genes start with codon ATG. As for the terminal codon, three (TAG for *ND3* gene, AGG for *ND6* gene and TAA/T for the rest) are used. Both 12S rRNA (952 bp) and 16S rRNA (1701 bp) genes are between tRNA-*Phe* and tRNA-*Leu1* genes, separated by tRNA-*Val* gene. Twenty-two tRNA genes interspersed between the rRNA and protein-coding genes, ranging from 67 (tRNA-*Cys*) to 74 bp (tRNA-*Leu1* and tRNA-*lys*). Except for the tRNA-*Ser2* replacing the dihydrouridine arm by a simple loop, the remaining can fold into a typical clover-leaf secondary structure. The control region (865 bp) is located between the tRNA-*Pro* and tRNA-*Phe* genes, with the rich A + T (63.6%) and poor G (14.7%) content.

In the Bayesian phylogenetic tree, all nodes are strongly supported (Figure 1). Seven monophyletic families are presented, and the main basal division is between clade (Berycidae + Holocentridae) and remaining families among which the family Diremidae is the basal clade and it also has a higher evolutionary rate than others. Within the family Holocentridae, it is clear that *S. spiniferum* is sister to *S. rubrum* that belongs to the same genus *Sargocentron* of *S. spiniferum* in morphological aspect.

**Figure 1.**
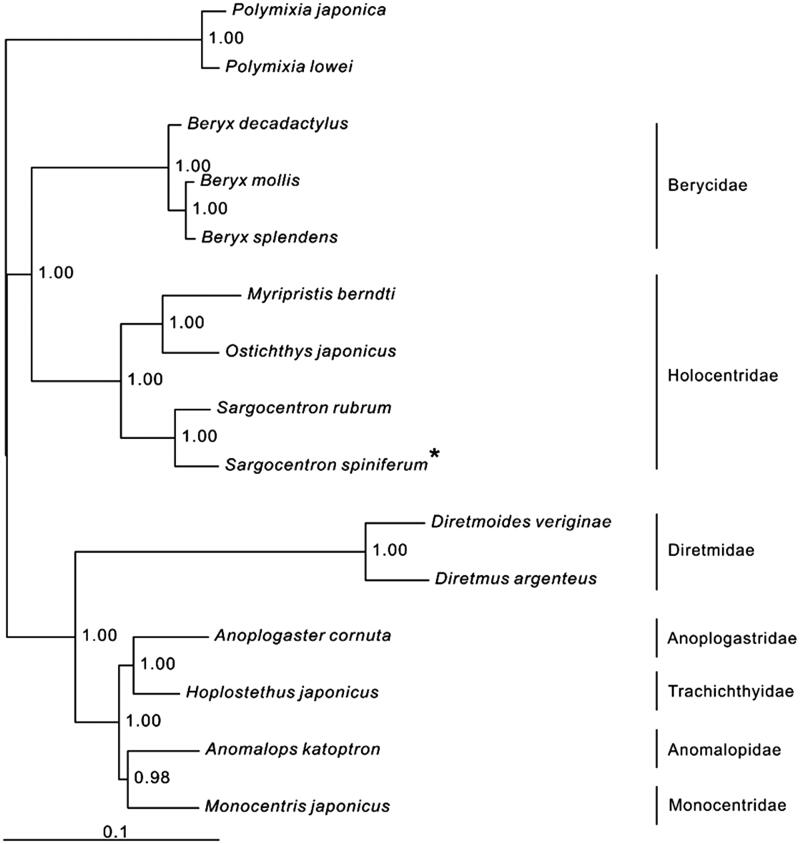
Phylogenetic position of *Sargocentron spiniferum Polymixia japonica* (AB034826.1) and *Polymixia lowei* (AP002927) were selected as the out group. The thirteen species from the order Beryciformes were: *Beryx decadactylus* (AP004430), *B. mollis* (DQ993168), *B. splendens* (AP002939), *Sargocentron spiniferum* (KX254549), *S. rubrum* (AP004432), *Myripristis berndti* (AP002940), *Ostichthys japonicus* (AP004431), *Anomalops katoptron* (NC_008128.1), *Anoplogaster cornuta* (AP004425), *Diretmoides veriginae* (AP004426), *Diretmus argenteus* (NC_008127.1), *Monocentris japonicus* (AP004429), *Hoplostethus japonicus* (AP002938).
